# Molar incisor hypomineralization and oral health-related quality of life: a sample of 8–12-years-old children

**DOI:** 10.1007/s00784-024-05490-z

**Published:** 2024-01-20

**Authors:** Seyma Kisacik, Cansu Ozsin Ozler, Seval Olmez

**Affiliations:** Faculty of Dentistry, Department of Pediatric Dentistry, Hacettepe University, 06100 Altindag, Ankara Turkey

**Keywords:** Children, Dentin sensitivity, Molar hypomineralization, Quality of life

## Abstract

**Objectives:**

The study aimed to evaluate the impact of molar incisor hypomineralization (MIH) and tooth sensitivity on the oral health in terms of the quality of life (OHRQoL). In addition, the impact of tooth maturity on tooth sensitivity was evaluated in the study.

**Materials and methods:**

Children aged 8–12 years with and without MIH participated in this descriptive cross-sectional study. They were chosen through the convenience sample technique. The *Pediatric-Oral-Health-related-Quality-of-Life (POQL)* scale was used to assess OHRQoL. The presence of the MIH and decayed, filled and missing teeth due to caries (*using dmft/s, DMFT/S indexes*) were recorded. The tooth sensitivity and dental maturity status were evaluated with the *Shiff-Cold-Air-Sensitivity-Scale (SCASS)* and *Demirjian-method*, respectively. Statistical analysis of the data was performed by Pearson Chi-Square Test and Mann-Whitney *U* test (*p*<0.05).

**Results:**

In this study, the participants were a total of 260 children, half were affected by the MIH. Their mean POQL scores were higher than those of the children without MIH with a statistically significantly difference in the total child scale score (*p*=0.014). Among the children with the MIH, child total QoL score was found to be higher in SCASS positive response group (*p*=0.011). The teeth with MIH (*p*<0.001) showed higher response to the stimulus. According to the dental age and dental maturity categories of the children with MIH, the total child scale score was found to be higher in the SCASS category (*p*=0.011), and the response status to the SCASS was statistically significant (*p*=0.042 and *p*=0.05, respectively).

**Conclusions:**

Among the children with MIH, the OHRQoL was found to be negatively affected. The teeth with the MIH tend to reveal more tooth sensitivity than healthy teeth. Many conditions such as having MIH, and tooth sensitivity might have an impact on the OHRQoL. In addition, more sensitivity to the stimulus was observed in the teeth with lower tooth maturity status; the dental age and dental maturity might have effects on tooth sensitivity.

**Clinical relevance:**

Considering the negative impacts due to MIH, the evaluation of OHRQoL is critical for dentists to employ a well-defined guide in their clinical decisions.

## Introduction

Molar incisor hypomineralization (MIH) is defined as a qualitative enamel defect of systemic origin, of which the etiology is unknown, affecting at least one permanent first molar and often including permanent incisors [1]. Affected teeth by the MIH have clinically visible hypomineralized areas; where the thickness of enamel is normal, but an abnormal enamel texture is observed due to a decrease in mineral content and increase in protein and water content. Enamel defects are seen as small, well-defined color changes or covering the entire surface of the tooth [2]. The color of hypomineralized enamel can vary from white to yellow or brown [3]. The porous enamel can easily breakdown, especially under the chewing forces. In rare cases, the enamel of the affected molars destroys soon after the eruption by leaving the dentin exposed, which is called “Posteruptive-Enamel-Breakdown (PEB)” [3, 4].

It is known that many conditions, such as increased interprismatic spaces in enamel, increased pulp innervation, and more immune cells presentation, increase the tooth sensitivity in affected teeth with MIH [5, 6]. Depending on the sensitivity, difficulties in brushing, poor oral hygiene risk, and eating difficulties which may affect the child’s diet and growth, may occur. Teeth affected by the MIH and with enamel disintegration or atypical restoration show more hypersensitivity [7]. In addition, rapid caries formation is observed in teeth with MIH [3], which causes various aesthetic problems [4, 8] and negatively affects the “Oral Health-related Quality of Life (OHRQoL)” of children and also, of parents [9–12]. According to the results of a recent systematic review and meta-analysis study [13] evaluating the relationship between MIH and OHRQoL, it was noted that OHRQoL was negatively affected. In addition, another recent systematic review [14] reported that OHRQoL was affected 17–25 times more in children with MIH than in children without MIH. It has been reported that when the teeth with excessive substance loss were restorated, the sensitivity does not completely disappear, but decreases, and the quality of life increases accordingly [15].

It is reported that a tendency to sensitivity, plaque accumulation, enamel destruction and dental caries formation increases with the age of children [16]. On the other hand, in the study of Linner et al. [7], it is stated that the patients have more sensitivity problems in permanent first molars with MIH in younger age group, and that sensitivity problems decrease with increasing age. It has been suggested that this may be due to the maturation stages of the tooth, such as physiological dentin deposition.

Although the relationship between MIH and tooth sensitivity has been known for a long time, there are no studies comparing the quality of life (QoL) with the tooth sensitivity. The aim of this study is to evaluate the OHRQoL among children regarding the MIH status and tooth sensitivity. In addition, the tooth sensitivity status among children with MIH with deference to the dental maturity and dental age were also tested in the study.

## Materials and methods

### Ethical concerns and design

**Fig. 1:**
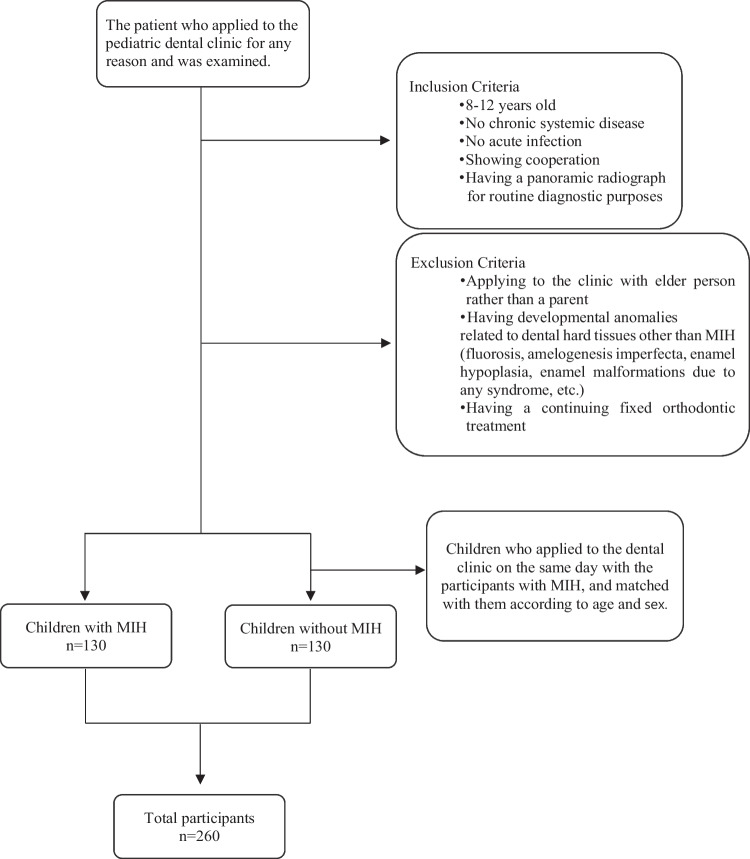
Sampling flow chart

In this descriptive cross-sectional study, MIH and QoL of the children were evaluated in accordance with the Declaration of Helsinki. The study was conducted at the Pediatric Dentistry Department of Faculty of Dentistry, Hacettepe University, between March 2020 and December 2021 after taking the approval from Hacettepe University’s Non-Interventional Clinical Research Ethics Committee (2020/03-24) and written consent of the participants’ parents. The participants were children aged 8–12 years who applied to dental clinic for any reason. In addition, they were included in the study if they did not have any chronic systemic disease or acute infection, and they should have a panoramic radiograph for diagnostic purposes. They were also required to take part in the study on a voluntary basis. Those children who did not apply to the clinic with their parents or had developmental anomalies related to dental hard tissue (fluorosis, amelogenesis imperfecta, enamel hypoplasia, enamel malformations due to any syndrome, etc.) other than MIH or received fixed orthodontic treatment were not chosen as the participant of the study. On the other hand, children with no MIH who applied to the clinic on the same day as participants with the MIH, were also included in the study. They were matched with those with MIH in terms of age and sex. In the study, the evaluations were carried out in two groups, children with MIH and without MIH (Fig. 1).

### Questionnaire and Oral Health–Related Quality of Life

The data of the study were collected using a questionnaire, the OHRQoL scale and through the oral examination and the evaluation of available panoramic radiographs. The questionnaire, consisting of demographical items, was administered to the parents in a face-to-face manner by the authors. The *Pediatric Oral Health-Related Quality of Life (POQL) scale* was used to evaluate the OHRQoL of the participants. This scale was developed by Huntington et al., to evaluate the OHRQoL among children aged 8–14 years, collecting data from the perspective of both the child and the parent [17]. Its validity and reliability in Turkish were found by Yazıcıoğlu et al. (Turkish-POQL) [18]. In this scale, there are seven general and ten scale items asked to the parents about their children. There are also six general and ten scale items asked to children. Of them, ten scale items are put under three sub-dimensions: role and physical function (four items), social function (three items) and emotional function (three items). Each of the scale items asks the frequency of an event and how the parent and children were disturbed from this situation. The scoring of the answers to these items are as follows: all of the time (score 3), some of the time (score 2), once in a while (score 1) or never (score 0). The scoring of the answers to the items about how disturbing the event is as follows: very bothered (score 4), somewhat bothered (score 3), bothered a little bit (score 2), never bothered (score 1) or didn’t happen (score 0) (Table 1). The answers stating “I don't know” were not included in the scoring. For each scale item, the scores to the answers to the items including the expressions “how often” and “how bothered” are multiplied in the process. The total score obtained is multiplied by 100 and divided by the maximum score that can be obtained from the scale. According to the Turkish-POQL, the three sub-scores were calculated separately, for the role and physical function, social function and emotional function based on the answers of both parents and children. Then the total scale score including all categories were calculated. To prevent the parent and children from being influenced by each other, the scale items were asked separately for both groups. The Turkish-POQL scores range from 0 to 100, and higher scores indicate that the child’s OHRQoL is negatively affected [18].

**Table 1:** The POQL scale questions and answer scores

Scale Questions (10 scale questions)	1-Pain 2-Eat hard food 3-Pay attention 4-Miss school 5-Not smile/laugh 6-Worry less attractive 7-Unhappy with looks 8-Angry/upset 9-Worry 10-Cry
How often the event occurred? (Score 0-3)	• All of the time (Score 3) • Some of the time (Score 2) • Once in a while (Score 1) • Did not happen (Score 0)
How bothered the parent or child was by its occurrence? (Score 0-4)	• Very bothered (Score 4) • Somewhat bothered (Score 3) • Bothered a little bit (Score 2) • Never bothered (Score 1) • Did not happen (Score 0)

### Intraoral examination

All children were examined intraorally by the same dentist (S.K.) under dental unit light using a dental mirror and ball-ended explorer. Carious status of the participants was evaluated by using the DMFT(S)/dmft(s) indexes [19]. Based on the definition of MIH [1], permanent first molars and incisors were examined. The teeth diagnosed with MIH were classified according to the criteria of the European Academy of Paediatric Dentistry (EAPD) [20] as follows; demarcated opacities, yellow and brown discolorations; enamel destruction and/or atypical caries after eruption; atypical restoration; molar extraction and non-eruptive teeth [20]. For the teeth with the PEB, it was thought to be together with “atypical caries” and categorized accordingly [21].

The severity of the MIH was grouped under two categories: 1— “Mild MIH” category (teeth with demarked opacity, yellow or brown discoloration); 2— “Severe MIH” category (enamel destruction and/or atypical caries after eruption; atypical restoration; molar extraction). In this categorization, it is assumed that if the child has a severe MIH in any of his/her teeth, it is considered as a severe case. If the child has a mild MIH in any teeth and no severe MIH findings, it is accepted as a mild MIH case [22, 23].

### Tooth sensitivity assessment

Personal pain experience was tested using the “Shiff Cold Air Sensitivity Scale (SCASS)” [24] to measure the tooth sensitivity. Among children with MIH, only the teeth affected by MIH were evaluated, whereas in the children without MIH, all existing permanent first molars were evaluated. The sensitivity test was not performed in the following cases: tooth performed or planned root-canal-treatment, extracted or planned extraction, with totally crown destruction, incompletely erupted, and with recently topical fluoride application (including SDF procedure). During this application, an air stimulus is applied for 1–2 s at a distance of 1 cm from the facial surface of the tooth. After the air stimulus was applied and the SCASS data were recorded, the stimulus responsiveness was evaluated both by considering the evaluated teeth and considering only the patients. The scoring of the patient’s responses to air stimulus is as follows: 0 = no response to the stimulus; 1 = have response to the stimulus, but no desire to move away from the stimulus; 2 = have response to the stimulus and move away from the stimulus; and 3 = have response to the stimulus, move away from the stimulus, and request immediate discontinuation of the stimulus. For statistical testing which is shown in the results part, a categorization was planned and if the patient/tooth did not respond to the stimulus, this was recorded as no response to the stimulus (score = 0), for the patient/tooth. Similarly, if the patient/tooth responded to the stimulus (score 1, score 2, score 3), this was recorded as a response to the stimulus for the patient/tooth.

### Panoramic radiograph and dental maturation assessment

According to the panoramic radiographic examination, maturation status of the teeth was evaluated. The method developed by Demirjian et al. [25] was used in this study. The morphological stages of seven teeth in the left lower jaw were investigated through the radiographs, and the age was determined by obtaining a maturity score. The findings were reported by comparing the SCASS response status of the children with MIH categorizing the dental maturity score between 67.3–93.1 and 93.2–99.2. The dental age was categorized between 7.6–10.7 and 10.8–15.8 years. These categories were organized based on the mean dental maturation score and dental age of all children.

### Statistical analysis and examiner information

A statistical analysis was performed using the Statistical Package for the Social Sciences Version 20.0 (SPSS Inc., Chicago, IL, USA). The data processing and statistical analysis were performed by the authors (SK and COO). One of them is an epidemiologist and a pediatric dentist (COO). The descriptive statistics was performed to produce the frequency and percentage for categorical variables, mean, median, minimum, and maximum values, the standard deviation, first and third quartiles, and interquartile range (IQR) for numeric variables. The Pearson chi-square and Fisher’s exact tests were used to test the difference between the independent categoric variables. The Mann–Whitney *U* test was performed to determine the median values of independent groups. The statistical significance value was set at *p*<0.05.

The collection of the data was performed by a research assistant (SK) of pediatric dentistry, who had taken training exercises by an experienced pediatric dentist and a professor. Inter and intra-rater agreements were determined based on the evaluation of the data obtained from twenty children who did not participate in the main study (the inter-rater Kappa coefficients were found to be 0.96 for the MIH detection; 0.84 for SCASS; and 0.98 for DMF index system). An intra-rater agreement was determined based on the data collected on two periods with a 7-day interval. The intra-rater kappa values for all scales were found to be higher than 0.80.

## Results

A total of 260 children, consisting of equal number of with and without MIH, was participated in the study. The age ranges of the participants and their dmft/s DMFT/S scores are shown in Table 2.

**Table 2:** Age and dmft/s-DMFT/S score distributions according to MIH presence

**Age and dmft/s -DMFT/S score**	**Children with MIH**		**Children without MIH**		***p*** ^**a**^
	***n* (130)**	**%**	***n* (130)**	**%**	
**Age (month)**					**0.049**
96–119	67	51.5	57	43.8	
120–155	63	48.5	73	56.2	
	X±SD=119.62±16.25		X±SD=123.73±17.23		
**dmft/s and DMFT/S score**	**Children with MIH**		**Children without MIH**		***p*** ^**b**^
	**Med**	**IQR**	**Med**	**IQR**	
dmft	3.5	1–6	4	4	0.330
dmfs	6.5	1.75–14	10	10	0.202
DMFT	3	2–4	3	3	0.052
DMFS	5	3–8	3	3	**<0.001**

*IQR: Interquartile range*

^***a***^*t test*

^***b***^*Mann-Whitney U test*

A total of 520 first permanent molars were evaluated among children with MIH. Of them 391 (75.2%) teeth showed instances of MIH. Of the teeth with MIH, 51.92% had demarcated opacity, yellow or brown discoloration, 36.7% of them had PEB and/or atypical caries, and 9.46% had atypical restorations. These findings indicate that thirty-two of the 130 participants with MIH had a mild MIH, while the remaining 98 had severe MIH. In addition, it was observed that OHRQoL scores of the child role-physical and emotional function sub-dimensions and total child dimension, were statistically significantly higher in children with severe MIH than that of the children with a mild MIH (*p*<0.05) (Table 3).

**Table 3:** Distribution POQL scores according to presence of MIH and MIH severity

**Scale sub-dimensions**	**POQL Score**				
	**Children with MIH (*n*=130)**		**Children without MIH (*n*=130)**		p*
	**Med**	**IQR**	**Med**	**IQR**	
**Parent sub-dimensions**					
Role and physical	16.67	8.33–29.69	12.5	4.17–29.17	0.060
Social	0	0–16.67	0	0–16.67	0.679
Emotional	22.22	8.33–45.14	22.22	5.55–41.67	0.413
Parent total	16.67	7.29–32.5	15	6.46–29.79	0.282
**Child sub-dimensions**					
Role and physical	18.75	12.5–31.31	12.5	4.17–25	**<0.001**
Social	5.55	0–22.92	0	0–16.67	0.178
Emotional	16.67	5.55–36.81	13.89	2.08–27.78	**0.037**
Child total	15	8.33–31.67	12.5	5–22.5	**0.014**
	**Mild MIH (*n*=32)**		**Severe MIH (*n*=98)**		p*
	**Med**	**IQR**	**Med**	**IQR**	
**Parent sub-dimensions**					
Role and physical	13.19	4.17–22.92	18.75	8.33–35.94	0.10
Social	0	0–12.50	0	0–17.36	0.98
Emotional	16.67	6.94–48.61	25	8.33–45.14	0.41
Parent total	15	5.21–28.75	17.08	7.50–32.71	0.68
**Child sub-dimensions**					
Role and physical	13.54	4.69–22.40	20.83	12.50–33.33	**0.04**
Social	0	0–11.11	8.33	0–30.56	0.15
Emotional	8.33	0–22.22	22.22	8.33–42.36	**0.014**
Child total	9.17	4.37–15.56	19.17	8.96–34.37	**0.003**

*IQR: Interquartile range*

*Mann-Whitney U test

When the OHRQoL of children were compared according to age groups (8–9 years and 10–12 years), no statistical difference was observed between them (*p*>0.05). The relation between dmft/s and DMFT/S scores and the POQL total scores of children and parents in our study was evaluated. While no correlation was found between dmft/s and QHRQOL (*p*>0.05), a statistically significant correlation was found between DMFT/S and OHRQoL in child and parent dimensions (*p*<0.05). Based on these findings, it seems that caries-related conditions in permanent dentition may affect OHRQoL.

Table 3 shows the distribution statistics of the POQL sub-dimension and total scale scores on the self-reports of the participants in regard to their MIH status. It was observed that the mean total and sub-dimensional scale scores were higher in children with MIH showing higher negative impacts. However, a statistically significant difference between the two groups was observed in the total child scale score (*p*=0.014), role and physical function (*p* <0.001) and emotional function (*p*=0.037) scores.

The findings from the SCASS test applied to permanent first molars showed that 59.2% of children with MIH and 16.9% of children without MIH responded to the stimulus (Table 4). Among the children with MIH, the stimulus response percentages are found to be statistically significantly higher (*p*<0.001). Similarly, the SCASS scale results on the permanent first molar teeth among children with MIH were statistically significantly more sensitive than those about the teeth in children without MIH group (p<0.001) (Table 4).

**Table 4:** Distribution of stimulus response status according to the presence of MIH of the children/teeth

**SCASS response status**	**Children with MIH**		**Children without MIH**		p*
	***n* (130)**	**%**	***n* (130)**	**%**	
**Patient**					**<0.001**
No response to stimulus	53	40.8	108	83.1	
Response to stimulus	77	59.2	22	16.9	
	***n* (366)**	**%**	***n* (509)**	**%**	
**Teeth**					**<0.001**
No response to stimulus	227	62.2	482	94.5	
Response to stimulus	139	37.8	27	5.5	

*Pearson Chi-square test

Table 5 presents the distribution statistics of the POQL scale and the total and sub-dimension scores according to the stimuli response status (“have response” or “no response”) of children with MIH. It is observed that the POQL sub-dimension and total scores of the patients who responded to the stimulus and those of their parents were higher than the patients who did not respond to the stimulus. A statistically significant difference was found in terms of the children’s self-reported role, physical function (*p*=0.006) and the total scale score (*p*=0.011).

**Table 5:** Distribution statistics of POQL scores according to stimulus response status in the children with MIH

**Scale sub-dimensions**	**SCASS response status**				
	**No response (*n*=53)**		**Having response (*n*=77)**		p*
	**Med**	**IQR**	**Med**	**IQR**	
**Parent total sub-dimension**	14.17	7.08–29.58	18.52	7.08–38.15	0.148
Role and physical function	16.67	7.29–28.12	18.75	8.33–36.46	0.146
Social function	0	0–16.67	0	0–18.05	0.373
Emotional function	19.44	9.72–36.11	22.22	8.33–58.33	0.275
**Child total sub-dimension**	12.5	5.83–25.42	19.17	9.17–35.17	**0.011**
Role and physical function	14.58	6.25–25	20.83	12.5–33.33	**0.006**
Social function	0	0–19.44	8.33	0–27.78	0.146
Emotional function	13.89	5.56±27.78	19.44	6.94–43.05	0.093

*IQR: Interquartile range*

*Mann-Whitney U test

The distribution of the stimulus response status of the children with MIH according to the dental maturity score and dental age categories is shown in Table 6. Stimulus responsiveness is more common among the children with a lower dental maturity score. This difference between the stimulus response status according to the dental maturity categories was found marginally statistically different (*p*=0.05). Comparison to the dental age categories revealed that the children with a lower dental age were more likely to respond to a stimulus. This difference between stimulus response status according to the dental age category was found to be statistically significant (*p*=0.042).

**Table 6:** Distribution of stimulus response status of children with MIH according to dental maturity score groups and dental age groups

**Dental maturity score and dental age**	**SCASS Response Status**				p*
	**No response**		**Response**		
	***n***	**%**	***n***	**%**	
**Dental maturity score**					0.05
67.3-93.1	26	49	51	66.2	
93.2-99.2	27	51	26	33.8	
**Dental age (year)**					**0.042**
7.6-10.7	20	37.7	43	55.8	
10.8-15.8	33	62.3	34	44.2	

*Pearson Chi-square test

## Discussion

In this study, our primary objective was to assess the OHRQoL in children concerning MIH status. Concurrently, our aim encompassed the evaluation of tooth sensitivity among children affected by MIH. Given the potential influence of factors such as enamel maturation, open root apices, and larger pulp chambers on sensitivity in teeth affected by MIH, we also sought to discern differences in tooth sensitivity between children with MIH and various dental maturity and dental age categories.

The assessment of caries in permanent teeth was conducted using the DMFT/S index in our study. The mean DMFT/S scores were observed to be higher in children affected by MIH, with a statistically significant difference noted for the DMFS scores. This outcome aligns with existing literature [26–29], demonstrating a positive association between enamel defects and dental caries, with a heightened prevalence of dental caries in children affected by MIH. In parallel with other clinical challenges associated with MIH, our study substantiates that MIH can adversely impact OHRQoL, encompassing both aesthetic and functional dimensions [9–12, 30, 31].

In previous studies, Turkish-POQL scale was employed to assess various oral health variables across different patient groups to evaluate OHRQoL [32–34]. This scale, whose Turkish version’s validity and reliability were established in 2018 [18], evaluates OHRQoL in children through self-reports from both the child and the parent. In comparison to other methods utilized for OHRQoL measurement, POQL was developed with a focus on the experiences and viewpoints of low-income families. Consequently, it is suggested that POQL may be a suitable scale, aligning with the oral and dental health profile identified by Gökalp et al. [35] in Turkish children [18]. However, there is no study using the patient with MIH considering the dental maturity. Nevertheless, there is currently no study utilizing the POQL scale specifically for patients with MIH while taking dental maturity into account. The results of POQL scale, including all sub-dimensions and total scale scores, were higher in children with MIH. This finding suggests a potential negative impact on OHRQoL in children affected by MIH. The observed difference between the two groups, as per the responses of the children, was found to be statistically significantly associated with the role-physical function, emotional function, and the overall scale score. Considering the challenges documented in several studies involving children with MIH [30, 31, 36], we observed a negative impact on OHRQoL within the sub-dimension referred to as the ‘role-physical sub-dimension’ in our study. This sub-dimension is commonly termed ‘oral symptoms and/or functional limitation’ in other studies. The notable finding identified in our study between the emotional sub-dimension (angry/upset, worry, cry), as reported by children, and MIH suggests that the impact of the role-physical sub-dimension may extend to emotional aspects for the children.

In the study conducted by Leal et al. [37], it was reported that the appearance of teeth in children with MIH negatively affects the perceptions of both parents and children. However, in our current study, we did not observe a significant difference in the OHRQoL within the social function sub-dimension (not smile/laugh, unhappy with looks) based on the perceptions of both the child and the parent. This finding is consistent with that of Portella et al. which reported no relationship between the perception of parents on this issue and MIH. Furthermore, as indicated by the findings from a recent systematic review and meta-analysis, OHRQoL was reported to be adversely impacted in the overall score and all sub-dimensions, with the exception of the social function sub-dimension [13]. Consistent with these results, it was reported that MIH did not lead to situations such as social isolation or desire to withdraw from friends. However, it should be noted that in our study, an OHRQoL assessment regarding MIH involvement was not performed for the participants by including examination of the anterior teeth. However, although there is no evidence that permanent molars with MIH do not contribute to aesthetic concerns, our study revealed that MIH generally does not have a negative impact on the social function subdimension. Additionally, contradictory findings may arise due to the impact of cultural differences on aesthetic perceptions.

In this study, it was observed that MIH has a more pronounced impact on the child than on the parent. The questions assessing “pain, eating hard food, paying attention, missing school,” within the role-physical function sub-dimension, are derived from the child’s individual experiences. Considering that the complications resulting from MIH are personally experienced by the child, it could be argued that the child is more significantly affected by this situation. Consequently, it is hypothesized that the parent may not perceive the negative impact to the same extent as the child experiences it. This observation aligns with the findings reported in the study by Arrow et al. [38], which specifically investigated the connection between developmental enamel defects in children and OHRQoL solely through parental perspectives and concluded that no significant relationship existed.

The maturity scores of the teeth in children with MIH were obtained and assessed based on the SCASS scores. Regarding both dental maturity score and dental age, it was observed that with less mature teeth and younger children were more prone to respond to stimuli. This discovery aligns with the results presented in the study by Linner et al. [7], which reported that higher tooth sensitivity is prevalent in younger patients, particularly those with newly erupting teeth.

It is reported that tooth sensitivity has a negative impact on the quality of life [6]. In this study, children with MIH who exhibited responsiveness to stimuli demonstrated higher POQL scores, both from their own perspective and as reported by their parents. In previous studies evaluating sensitivity [7, 39, 40], commonly used tests included the SCASS test along with other assessments that incorporated the patient’s subjective opinion. However, in our study, this evaluation was conducted exclusively using the SCASS test.

There are certain limitations to the study. The research commenced, and shortly thereafter, the coronavirus pandemic ensued, resulting in the cancellation of all procedures except for emergency treatments. This situation led to a significant decrease in the number of patients applied dental services during the study period. Another limitation of the study was the use of a convenience sample technique in participant selection. Despite this, it is notable that the number of patients included in our study closely aligns with the participant numbers in other recent studies employing a similar study design [10, 12, 30, 41].

Considering the adverse effects of tooth sensitivity on OHRQoL and the variations in sensitivity across dental maturity and age categories, it is believed that the findings of this study may provide valuable guidance for future research. Given that OHRQoL can be influenced by numerous factors, our findings should be assessed with consideration for these factors. The reported findings should be evaluated within the limitations of the study design. Therefore, the OHRQoL of children with MIH should be routinely assessed, and current preventive and/or restorative treatments should be applied based on these evaluations.

In conclusion, high OHRQoL scores indicating negative impact were detected in children with MIH. Additionally, it was observed that the OHRQoL of children with MIH who experienced tooth sensitivity was more adversely affected. Moreover, it was noted that sensitivity to stimuli increased in teeth with lower tooth maturation status.
